# Effectiveness of Telecoaching and Music Therapy in Neurological Disorders: A Narrative Review and Proposal for a New Interventional Approach

**DOI:** 10.3390/healthcare13070826

**Published:** 2025-04-04

**Authors:** Ignazio Leale, Claudia Vinciguerra, Vincenzo Di Stefano, Filippo Brighina, Giuseppe Battaglia

**Affiliations:** 1 Sport and Exercise Research Unit, Department of Psychology, Educational Sciences and Human Movement, University of Palermo, 90144 Palermo, Italy; giuseppe.battaglia@unipa.it; 2 Neurology Unit, Department of Medicine, Surgery and Dentistry “Scuola Medica Salernitana”, University Hospital “San Giovanni di Dio e Ruggi D’Aragona”, 84131 Salerno, Italy; claudia.vinciguerra@sangiovannieruggi.it; 3 Department of Biomedicine, Neuroscience, and Advanced Diagnostics (BiND), University of Palermo, 90129 Palermo, Italy; vincenzo.distefano07@unipa.it (V.D.S.); filippo.brighina@unipa.it (F.B.)

**Keywords:** neuromuscular disorders, music therapy, telecoaching, quality of life, health promotion

## Abstract

Neurological disorders represent a significant challenge for healthcare systems, necessitating innovative and multidisciplinary therapeutic approaches. These disorders often lead to difficulties in various aspects of daily life, including physical functioning, mental health, and quality of life (QoL). Telecoaching (TC) and Music Therapy (MT) are two emerging modalities that may provide valuable support for individuals with these conditions. This narrative review aims to analyse recent studies investigating the efficacy of TC and MT in this population. A total of 23 studies were included in this narrative review. These studies indicate that TC positively affects physical functioning and adherence to intervention programs, with participants reporting improvements in gait and balance, as well as a reduction in postural instability and fall rates. Similarly, MT has shown promising outcomes in decreasing anxiety and sleep disorders while enhancing cognitive and emotional well-being. Integrating TC and MT into treatment approaches offers a promising alternative for improving QoL and overall well-being. However, further research is needed to consolidate the evidence and optimize the implementation of these interventions in clinical practice. Future research should focus on longitudinal studies and comparative evaluations to further clarify the role of TC and MT in the treatment of neurological disorders, as well as their long-term effects.

## 1. Introduction

A study published in the Lancet Neurology as part of the Global Burden of Disease research found that in 2021, more than three billion people, equivalent to 43% of the global population, were affected by neurological conditions. This reflects an 18.2% increase compared to 1990 and a 1.5 increase over the past two decades [1]. Neurological disorders, including neurodevelopmental conditions and diseases affecting the nervous system, were identified as the leading cause of disability worldwide [1], accounting for a total of 168 million years lived with disability. Among the conditions examined were multiple sclerosis (MS), Parkinson’s disease (PD), Alzheimer’s disease (AD), and migraine, all of which present significant challenges for both patients and healthcare professionals due to their complexity and substantial impact on Quality of Life (QoL) [2,3]. The recent World Health Organization’s Action Plan outlines a comprehensive strategy based on five objectives to improve brain health worldwide [4]: (a) increasing policy priority; (b) providing effective, timely, and responsive diagnosis, treatment, and care; (c) implementing promotion and prevention strategies; (d) promoting research and innovation; and (e) strengthening the public health approach. This plan emphasizes the importance of collaboration among stakeholders in the health sector and beyond, promoting the involvement and empowerment of individuals with neurological disorders, along with their caregivers and families [5]. In recent years, there has been a growing interest in integrating innovative therapeutic approaches for the treatment of neurological disorders [6]. The COVID-19 pandemic necessitated significant adaptations in healthcare practices, leading professionals to comply with social distancing guidelines and quarantine protocols. As a result, many transitioned from in-person consultations to virtual modalities, accelerating the adoption of telemedicine interventions [7]. In this context, non-conventional therapeutic approaches, such as telecoaching (TC), are gaining attention for their potential to enhance patients’ QoL and overall well-being. TC is a new intervention approach that utilizes digital technologies, such as a phone, personal computer, or tablet, to provide personalized support and continuous monitoring, enabling individuals to access training programs remotely [8]. This innovative approach can improve treatment adherence, enhance self-efficacy, and promote greater involvement in health management.

Another important emerging approach is music therapy (MT), which involves the clinical and evidence-based use of music interventions to achieve individualized therapeutic goals, addressing physical, emotional, mental, social, and cognitive needs within a structured therapeutic relationship [9,10,11,12], either “in person“as well as in “remote and virtual” mode (through telemedicine) [13]. MT has been shown to improve overall benefits for QoL and well-being, and it positively influences the nervous system by modulating the parasympathetic, dopaminergic, and opioid receptors [14].

The application of TC and MT can be particularly significant for neurological patients, who often experience social isolation and heightened anxiety [15,16]. Indeed, the remote accessibility of these therapies helps to overcome geographical and logistical barriers, which is essential for individuals with mobility challenges, a common issue among neurological patients [17]. Ultimately, these innovative approaches may be more sustainable and cost-effective in the long term, potentially reducing the financial burden associated with conventional treatment modalities.

Thus, this narrative review examines the use and effectiveness of TC and MT in individuals with neurological disorders, analysing the available evidence. Through a critical synthesis of the literature, the review aims to develop practical recommendations for health and exercise professionals, highlighting implications for practice and future directions. Additionally, this study proposes a strategic design for interventions that integrate TC and MT. The integration of TC and MT highlights their complementary nature, offering a more comprehensive approach to patient care. This integrated perspective can enhance treatment outcomes by simultaneously addressing both the physical and emotional needs of patients.

## 2. Methods

This narrative review was developed in accordance with the Assessment of Narrative Review Articles (SANRA) guidelines [18]. It analyses the existing literature on the understanding, management, and treatment of neurological disorders, with a particular focus on non-pharmacological approaches such as TC and MT. Specifically, it explores their mechanisms of action, clinical applications, and potential benefits in enhancing quality of life and overall well-being in individuals with neurological conditions. Eligible articles were identified through a manual search of the following databases: PubMed, Scopus, and Web of Science. The search was conducted up to 10 January 2025. The following terms and keywords were used in combination with the Boolean operators “AND” and “OR”: “music therapy”, “musicotherapy”, “telecoaching”, “telehealth”, “home exercise”, “physical activity”, “exercise”, “neurological disorders”, and “neurological diseases”. To ensure comprehensive coverage, additional relevant articles were identified by screening the reference list of selected studies. Only peer-reviewed, English-language articles focusing on the recent advancements in the understanding and treatment of neurological diseases were included. Eligible studies encompassed randomized controlled trials, non-randomized controlled trials, and observational studies. Articles that did not provide information on TC programs or MT interventions were excluded. The selected studies were analysed through a qualitative synthesis approach. Key findings were categorized based on intervention type, study design, target population, and main outcomes. The findings are presented in structured subsections, each addressing key aspects of TC and MT intervention, including their effectiveness, mechanisms of action, and potential clinical implications.

## 3. Results

In recent years, numerous studies have investigated innovative strategies to promote healthy behaviours in individuals with neurological disorders, offering promising advancements in treatment and care. Among the most notable emerging interventions are TC and MT, both of which have shown encouraging results in improving health outcomes and QoL in this population.

### 3.1. Telecoaching and Neurological Disorder

#### 3.1.1. Telecoaching and Parkinson’s Disease

PD is one of the neurological disorders in which the TC approach has been most applied [19]. Several studies have assessed the effectiveness of TC-based training programs in this population. One prominent study conducted by Gandolfi and Colleagues (2017) compared postural stability improvements following remotely supervised virtual reality (VR)-based home balance training with sensory integration-based balance training (SIBT) conducted in a clinical setting [20]. In this multicenter study, 76 patients with PD were randomly assigned to either the TC group or the SIBT group. The intervention program consisted of 21 sessions, each lasting 50 min, conducted three times a week for 7 weeks. In the TC group, each session began with a short warm-up, including stretching exercises for the upper and lower limbs. The central phase involved ten exercises tailored to the patient’s specific clinical condition, utilizing the Wii Fit and balance board. In the SIBT group, the training session also began with a short warm-up phase, followed by static and dynamic balance exercises performed under various sensory conditions. Each exercise was repeated five to ten times, depending on the patient’s abilities, with gradual increases in intensity and volume to promote improvement. Patients were assessed before and after the intervention, as well as at one-month follow-up, using the Berg Balance Scale (BBS), Activities-Specific Balance Confidence, 10-Meter Walking Test, Dynamic Gait Index, Parkinson’s Disease Quality of Life questionnaire (PDQ-8). The number of falls in the previous month was also recorded. Additionally, a cost analysis was conducted to compare the two approaches. The results showed a significant improvement in BBS scores in the TC group (*p* = 0.04) and a significant Time × Group interactions effect in the Dynamic Gait Index for the SIBT group (*p* = 0.04). Both groups demonstrated improvements in all outcome measures over time, except for fall frequency. The cost analysis revealed that the TC group incurred lower treatment and equipment costs compared to the SIBT group.

Yang and Colleagues (2016) also investigated the effectiveness of TC using VR in comparison to conventional balance training in individuals with PD [21]. A total of 23 participants were randomly assigned to either the TC group (*n* = 11) or the control group (*n* = 12). Both groups completed 12 training sessions, each lasting 50 min. The TC group engaged in VR-based exercises combined with a balance board, focusing on postural maintenance and dynamic tasks that simulated daily activities. In contrast, the control group participated in conventional, therapist-supervised training, which included static and dynamic exercises aimed at improving balance. The primary outcome measure was the BBS, while secondary outcomes included the Dynamic Gait Index, the Timed Up-and-Go (TUG) Test, the Parkinson’s Disease Questionnaire (PDQ-39), and the motor score of the Unified Parkinson’s Disease Rating Scale. Assessments were conducted at baseline, after the training program (6 weeks, post-test), and at week 8 (follow-up). Both groups showed improvements across all outcome measures compared to baseline. However, no statistically significant differences were observed between the two groups at either the post-test or follow-up assessments.

Atterbury and Welman (2017) employed a TC approach to evaluate the effectiveness of a home-based balance training program compared to a conventional therapist-supervised training program in individuals with mild to moderate PD [22]. A total of 40 participants were divided into two groups: the TC group (*n* = 16), which performed home exercises, and the control group (*n* = 24), which engaged in therapist-supervised sessions. Both groups completed 8 weeks of training, with three sessions per week, each lasting between 40 and 60 min. Each session comprised a 10-min warm-up, 15–40 min of balance exercises, and 10 min of cool-down. Outcome measures included the Timed-Up-and-Go Test, Functional Gait Analysis (FGA), Activity-Specific Balance Confidence (ABC) Scale, and Intrinsic Motivation Inventory (IMI). Both groups exhibited improvements in the FGA, with a significant increase in step length (*p* < 0.05). However, enhancements in the ABC Scale (*p* = 0.05), stride velocity (*p* = 0.0006), and cadence (*p* = 0.046) were observed only in the control group, which received therapist supervision. Additionally, the control group demonstrated significantly higher motivation levels (*p* = 0.002).

#### 3.1.2. Telecoaching and Multiple Sclerosis

Another neurological disorder in which the effectiveness of the TC approach has been investigated is multiple sclerosis (MS). An important study by Pagliari and colleagues (2016) evaluated the effectiveness of TC using VR compared to conventional rehabilitation intervention for patients with MS [23]. This multicenter, randomized, controlled trial involved 70 participants, with 35 assigned to the TC group and 35 to the control group. Both groups completed 30 training sessions, consisting of five sessions per week, each lasting 45 min. The TC group engaged in exercises using a dedicated VR Rehabilitation System Kit that incorporated digital cognitive and motor content, with asynchronous training monitored remotely by a therapist. In contrast, the control group received active treatment, which included conventional motor and cognitive exercises. The main outcomes assessed included the cognitive and psychological motor profiles of the participants, measured by the Multiple Sclerosis Quality of Life-54 (MSQOL-54), the Mini-Balance Evaluation System Test (Mini-BESTest), and the State-Trait Anxiety Inventory (STAI). The findings revealed that 63.3% of the TC group experienced improvements in the physical domain of QoL (*p* = 0.045). Furthermore, the TC group demonstrated significantly greater enhancements compared to the control group in the Mini-BESTest domains of balance (*p* = 0.014), postural control (*p* = 0.024), and dynamic walking (*p* = 0.020) at post-treatment. Additionally, higher adherence rates were observed in the TC group compared to the control group (86.67% vs. 80.0%).

Hoang and colleagues (2015) employed a TC approach to evaluate whether step training could improve physical and neuropsychological measures related to falls in individuals with MS [24]. This randomized controlled trial involved 50 patients with moderate disability, who were divided into two groups: a TC group (*n* = 28) and a control group (*n* = 22). Participants in the TC group engaged in two interactive exergames, participating in two 30-min sessions per week over 12 weeks. The first exergame utilized open-source software (Stepmani) (https://www.stepmania.com/) to create a rhythmic video game, synchronizing movements with visual stimuli displayed on a monitor. The second exergame focused on fast and precise movements involving both lower limbs. Conversely, participants in the control group continued with their usual physical activity. The primary outcomes of the study included Choice Stepping Reaction Time (CSRT) and Stroop Stepping Test (SST) time. Secondary outcomes included measures of balance (postural sway and CSRT components), gait speed, cognitive tests, and the Nine-Hole Peg Test (9-HPT). The TC group demonstrated statistically significant improvements in CSRT and SST, as well as in open-eye oscillation, the 9-HPT, and gait speed compared to the control group. Additionally, a non-significant trend toward fewer falls was observed in the intervention group. Importantly, no adverse events were reported during the intervention program.

Güngör and colleagues (2022) utilized a TC approach to evaluate the effectiveness of a home-based Pilates-based core stability training (PBCST) program on lower-limb strength and postural control in individuals with MS, comparing it to the same program conducted under supervision [25]. In this study, 50 individuals with MS were randomly assigned to two groups: a TC group, which performed the training program independently at home, and a control group, which completed the routine training program under supervision in a clinical setting. Both groups followed the training program for 8 weeks, participating in two sessions per week, each lasting approximately 60–75 min. Exercises were performed in different positions, including supine, lateral, prone, quadrupedal, sitting, and standing, and were complemented with self-stretching exercises during the final phase of each session. The primary outcomes of the study included postural oscillation and isokinetic muscle strength of the lower limbs, assessed using the Modified Clinical Test of Sensory Integration of Balance (m-CTSIB), the Balance Error Scoring System (BESS Test), the Postural Stability Test (PST), and a Biodex Multi-Joint System (isokinetic dynamometer). Secondary outcomes included physical ability and fatigue. Statistically significant improvements were observed in all parameters for both groups, although certain sub-parameters of postural oscillation in the TC group did not reach statistical significance (*p* < 0.05). Overall, the control group demonstrated slightly greater improvements.

#### 3.1.3. Telecoaching and Ataxia

Cabanas Valdés and colleagues (2024) utilized a TC approach to investigate the effectiveness of a home-based core stability exercise (CSE) program on ataxia severity, functional abilities, QoL, and fall rates in individuals with both short- and long-term hereditary ataxia [26]. This randomized controlled trial involved 23 participants, divided into two groups: a TC group that received standard care combined with the home-based CSE program and a control group that received standard care alone. Participants in the TC groups performed the CSE independently, engaging in sessions five days a week for 5 weeks, with each session lasting 60 min. The exercises were designed to strengthen trunk muscles and enhance proprioception, incorporating movements in various positions, including supine, sitting on an unstable surface, prone, and standing. Assessments were conducted at baseline, at the end of the intervention (5 weeks), and at follow-up (10 weeks). The primary outcomes included ataxia severity and trunk function, while secondary outcomes included the following: balance confidence, measured by the Activities-Specific Balance Confidence (ABC) Scale; gait speed, assessed with the 4-Meter Walk Test (4-MWT); lower-limb motor function, evaluated by the 30-Second Sit-to-Stand Test; QoL, measured using the EuroQol 5-Dimension 5-Level (EQ-5D-5L); health status, assessed with the EQ-5D Index and fall rates. Although no statistically significant differences were observed between the groups regarding ataxia severity or trunk function, significant improvements were noted in balance confidence, gait speed, QoL, and fall rates. A statistically significant interaction between groups and time was observed for the ABC Scale (*p* = 0.007), the EQ-5D-5L total score (*p* = 0.013), and the EQ-5D index (*p* = 0.006).

Jabri and colleagues (2022) conducted a study investigating how a home TC training program, with and without vibrotactile Sensory Augmentation (SA), influences balance and coordination in individuals affected by hereditary cerebellar ataxia [27]. This crossover study recruited 10 patients who participated in two blocks of training, each lasting six weeks. Participants were instructed to perform balance and coordination exercises five times a week for a total of 12 weeks, with each session lasting 30 min. The exercises were categorized into five groups: Static Standing, standing on a Compliant Surface, Arm Raises, Weight Shifting, and Gait. Regardless of whether they received vibrotactile SA, all participants utilized a smartphone balance trainer during their home training sessions. This balance training system included an Apple iPod, four tactor buds attached to an elastic belt, and a portable Apple iPod. During sessions incorporating vibrotactile SA, the tactors delivered directional vibrotactile feedback corresponding to the navel, lumbar spine, and right and left sides of the trunk. When a participant’s control signal exceeded a predefined threshold, the corresponding tactor was activated to deliver a vibrotactile signal, prompting participants to make postural corrections by “moving away from the vibration”. Participants were instructed to record any step-outs and self-evaluations using the iPod interface, with these data automatically uploaded to a secure cloud server for review by a physical therapist who remained unaware of the participants’ identities or group assignments. Each week, the therapist personalized exercises for each participant based on these data. Performance was assessed before, during, and after the training program. Several assessment measurements were used, including the Scale for the Assessment and Rating of Ataxia (SARA), the posture and gait sub-scores of SARA, the Dynamic Gait Index, the modified Clinical Test of Sensory Interaction in Balance, and the TUG Test. Among the seven participants who completed both training blocks, the changes in SARA scores and the SARA posture and gait sub-scores following training with SA were not statistically significant compared to those observed after training without SA (*p* > 0.05). However, a trend toward indicating improved SARA scores and sub-scores for posture and gait was observed with vibrotactile SA. Significantly, participants demonstrated improvements in their SARA scores (*p* = 0.02) and SARA posture and gait sub-scores (*p* = 0.01) when compared to their pre-training evaluations with vibrotactile SA.

#### 3.1.4. Telecoaching and Other Neurological Disorders

The TC approach was employed by Burns and colleagues (2017) to evaluate the safety and effectiveness of progressive resistance exercise for addressing foot dorsiflexion weakness in children with Charcot–Marie–Tooth disease [28]. In this randomized, double-blinded, controlled study, 60 children were assigned to either a progressive resistance training group (*n* = 30) or a control group receiving a sham training program (*n* = 30). All participants engaged in training three times a week for six months, focusing on dorsiflexion exercises for each foot, using an adjustable exercise cuff specifically designed to increase load capacity. For the exercise group, the intensity was initially set at 50% of one repetition maximum (1RM) and was gradually increased to 70% over the training period. In the control group, participants followed the same procedure but trained at a very low intensity (less than 10% of 1RM) without any increases throughout the training. The primary outcome was the between-group difference in dorsiflexion strength, expressed as a Z score, assessed using hand-held dynamometry at baseline and at 6 months, 12 months, and 24 months. The ANCOVA-adjusted Z score differences in dorsiflexion strength between groups were as follows: 0 (95% CI −0.37 to 0.42; *p* = 0.91) at 6 months, 0.3 (95% CI −0.23 to 0.81; *p* = 0.27) at 12 months, and 0.6 (95% CI 0.03 to 1.12; *p* = 0.041) at 24 months. Overall, the study concluded that six months of targeted progressive resistance exercise effectively attenuated the long-term progression of dorsiflexion weakness in paediatric patients with Charcot–Marie–Tooth disease, with no reported adverse events.

Mehta and colleagues (2021) utilized a TC approach to compare the effectiveness of physical therapy (stretching) and yoga therapy as complementary tools to standard drug treatment in patients with migraine [29]. A total of 61 participants were divided into three groups: a physical therapy group (*n* = 20), a yoga therapy group (*n* = 20), and a standard therapy group (*n* = 21). All participants followed their respective programs for 12 weeks: the physical therapy group practiced relaxation exercises, neck stretching, and cardio-respiratory resistance training; the yoga therapy group performed specific exercises, including the butterfly position (Bhadrasana), cobra position (Bhunjagasana), and foot touch (Padhastasana); and the standard therapy group continued only with drug treatment. The outcome measures assessed included headache frequency, the Short-Form McGill Pain Questionnaire (SF-MPQ), and the Headache Impact Test-6 (HIT-6). These assessments were conducted at recruitment and subsequently at monthly intervals for three months. All three groups demonstrated a significant reduction in headache frequency when comparing baseline measurements with post-intervention assessments at 1 month, 2 months, and 3 months (*p* < 0.05). However, the reduction in headache frequency was significantly greater in the physical therapy group compared to the other two groups. Furthermore, all three groups showed significant differences in sensory and affective pain ratings between pre-intervention and post-intervention at 1 month, 2 months, and 3 months. A one-way ANOVA Test revealed a statistically significant reduction in headache frequency across all groups at 3 months post-intervention.

Menengi and colleagues (2022) evaluated the effectiveness of home-based exercise treatment using a TC approach in patients with Alzheimer’s disease (AD) [30]. The study involved 20 individuals with early to middle-stage AD, who were randomized into two groups: a TC group (*n* = 10) and a control group (*n* = 10). Participants in the TC group completed 25 training sessions over 6 weeks, with each session lasting between 15 and 40 min. All sessions included dual-task motor and cognitive exercises, utilizing specific strategies to maintain participant engagement, such as singing and rhythm activities. The progression of exercises was facilitated by introducing new activities throughout the program. Primary outcomes included the Mini-Mental State Examination (MMSE), the TUG Test, and the Five-Times Sit-to-Stand Test. Secondary outcomes included the One-Leg Stance Test (OLST), Katz Activities of Daily Living Scale (Katz-ADL), Functional Independence Measure, Geriatric Depression Scale-Short Form, Beck Anxiety Scale, Zarit Caregiver Burden Inventory (ZCBI), and the Warwick Edinburgh Well-Being Scale. These outcomes were assessed at baseline and post-treatment. Results indicated a significant difference in the mean change between the groups, favouring the TC group for all primary and secondary outcomes (*p* < 0.05), except for the ZCBI and OLST (*p* > 0.05). No significant differences were observed in the comparison of primary outcome measures between the groups in the post-treatment results (*p* > 0.05). However, significant differences in all secondary outcome measures were noted in favour of the TC group (*p* < 0.05), except for the OLST, Katz-ADL, and ZCBI (*p* > 0.05). More information about TC in different neurological disorders can be found in Table 1.

### 3.2. Music Therapy and Neurological Disorder

#### 3.2.1. Music Therapy and Alzheimer’s Disease

AD is one of the neurological disorders where the MT approach has been widely employed. A notable study by Gómez Gallego and colleagues (2017) investigated the clinical improvement profile of individuals with mild to moderate AD who underwent MT [31]. In this study, 42 patients received MT for 6 weeks, participating in two weekly sessions lasting 45 min each. Each session included various activities, such as a welcome song, rhythmic accompaniments using hands or musical instruments, movement exercises with background music, songs and interpreters guessing games, and a farewell song. The sessions were conducted by two music therapists in carefully managed environments. Participants were assessed for cognitive, neuropsychiatric, and functional outcomes after three weeks and at the end of the study period, using the following scales: MMSE, Neuropsychiatric Inventory (NPI), Hospital Anxiety and Depression Scale, and Barthel Index. The results indicated statistically significant improvement in memory, orientation, and depression in both mild and moderate cases, as well as reductions in anxiety for mild cases and decreases in delirium, hallucinations, agitation, irritability, and language disorders in participants with moderate AD. Notably, the effects on cognitive measures were evident after just four sessions of MT.

Gómez-Gallego and colleagues (2021) conducted a follow-up study to compare the effects of two MT interventions with a control activity in individuals with AD [32]. In this randomized study, 90 participants were assigned to one of three groups: an active music intervention (AMI) group, a musical receptive intervention (RMI) group, and a control group. The MT intervention consisted of two weekly sessions over 12 weeks, each session lasting 45 min. The AMI group engaged in activities, including a welcome song, rhythmic and dance exercises, a music quiz, and a farewell song, all aimed at enhancing cognition, attention, movement synchronization, socialization, and mood. In contrast, the RMI group listened to pre-recorded playlists, with the therapist announcing the title and artist after each song and encouraging participants to share their feelings or memories. This group did not participate actively, focusing instead on mood improvement, anxiety reduction, and socialization. The control group watched nature videos without music. Outcomes were assessed before and after the intervention using the MMSE, NPI, Geriatric Depression Scale (GDS), Barthel Index, and Tinetti Scale. Results indicated that the AMI group showed significantly greater improvements in cognition, behaviour, and functional status compared to both the RMI and control groups. The RMI groups had a stabilizing effect on behavioural symptoms compared to the control group. Specifically, 85.7% of participants in the AMI group showed improvements in cognitive deficits, 92.9% in behavioural symptoms, and 46.4% in functional status, compared to the RMI group (11.8%, 42.9%, and 14.3%, respectively) and the control group (6.3%, 12.2%, and 17.1%, respectively).

Lyu and colleagues (2018) also investigated the effects of music therapy on cognitive function and mental well-being in patients with AD [33]. In this randomized trial, 298 individuals with mild, moderate, or severe AD were randomly assigned to three groups: the MT group (*n* = 100), the lyric reading group (*n* = 99), and a control group (*n* = 99). The MT group engaged in listening and singing their favourite songs, predominantly classical and relaxing music. The lyric-reading group focused on reading song lyrics without any melodic accompaniment, while the control group received no intervention. Both MT and lyric reading sessions were conducted for 3 months, twice a day, with each session lasting 30 to 40 min. All participants continued receiving routine medical treatment throughout the study. Cognitive functions, neuropsychological symptoms, and activities of daily living were assessed at baseline, three months, and six months using the MMSE, the NPI, and the Barthel Index. Results indicated that MT was more effective than lyrics reading in improving verbal fluency and alleviating psychiatric symptoms and caregiver discomfort in patients with AD. Stratified analyses reveal that MT enhanced memory and language skills in patients with mild AD while it effectively reduced psychiatric symptoms and caregiver discomfort in those with moderate or severe AD. However, no significant effect on activities of daily living was observed.

#### 3.2.2. Music Therapy and Parkinson’s Disease

Pohl and colleagues (2020) developed an MT approach to evaluate the effectiveness of group music intervention in individuals with PD [34]. In this randomized controlled study, 46 individuals with PD were assigned to either the MT group (*n* = 26) or a control group (*n* = 20). The MT intervention was conducted twice a week for 12 weeks, with each session lasting 60 min. Each session began with stretching exercises, followed by 50 min of Ronnie Gardiner Method, a rhythm-based program emphasizing auditory beat perception to improve postural control, gait, and cognitive functions such as memory. The sessions concluded with soft classical music. The primary outcome was performance on the TUG test while subtracting serial sevens, measuring the effect of cognitive demands on functional mobility (motor-cognitive dual-tasking). Secondary outcomes included cognitive function, evaluated with the Montreal Cognitive Assessment Scale (MoCA) and three parts of the Cognitive Assessment Battery (the Test Recall Test for immediate and delayed recall, the Stroop Color-Word Test, and the Symbol Digit Modalities Test), as well as dynamic balance assessed by the Mini-BESTest. Additionally, participants completed three questionnaires: the Falls Efficacy Scale International, the Freezing of Gait Questionnaire, and the Parkinson Disease Questionnaire 39-item Global Index Score. While no differences were observed between the groups in dual tasks performance, significant improvements were noted in favour of the MT group for the Falls Efficacy Scale (*p* = 0.001) and the Parkinson Disease Questionnaire-39 (*p* = 0.005). However, these differences were not maintained three months post-intervention.

Fodor and colleagues (2021) investigated the effects of a multimodal rehabilitation program that combined physical therapy and art therapy with music listening (MT group, *n* = 16) on the QoL in individuals with PD, compared to a control group that participated in the same rehabilitation program without music (*n* = 16) [35]. Both groups engaged in a multimodal intervention program six times per week for 2 weeks, with each session lasting approximately 2 h. Each session began with about 60 min of physical therapy activity, including warm-up exercises, aerobic activities, resistance training, and balance exercises, followed by around 50 min of art therapy. A 10-min break for socializing and rest was provided between the two activities. Only the MT group incorporated music into the intervention program. The musical preferences of this group were different, with 50% (16 participants) preferring pop music, 25% (8 participants) preferring classical music, and 25% (8 participants) preferring rock music. Following the completion of the rehabilitation program, participants in the MT group were encouraged to listen to their favourite music for 2.5 h each day during the following two weeks, in addition to their regular daily activities. Assessments were performed using the self-reported PDQ-39 both at the beginning of the multimodal program and one month after its initiation. The MT group showed significantly greater improvement in five of the eight domains measured by the PDQ-39 compared to the control group, specifically in ADLs, emotional well-being, social support, communication, and bodily discomfort.

Li and colleagues (2022) investigated the effectiveness of MT in addressing gait freezing in individuals with PD [36]. In this randomized controlled trial, 81 participants were randomly assigned to one of three groups: an MT group (*n* = 27), an exercise therapy group (ET, *n* = 27), or a control group (*n* = 27). Both the MT and ET groups participated in a structured training program five times per week for four weeks, with each session lasting 60 min. However, only the MT group incorporated music into the training program. The control group continued with the usual care and treatment. The training program consisted of exercises such as flat start walking, turning, narrow space walking, and step training. The study assessed comprehensive motor function and Freezing of Gait using the Freezing of Gait Questionnaire (FOG-Q). After four weeks, the MT group demonstrated significantly lower values for double support time, cadence, maximum knee flexion in the standing position, maximum hip extension, knee flexion moment in the standing position, overall motor function, and FOG-Q scores compared to both the control and ET groups (*p* < 0.05). Additionally, the MT group exhibited significantly higher gait speed, maximum ankle dorsiflexion in the standing position, ankle range of motion (ROM) during push-off and the gait cycle, knee ROM during the gait cycle, and maximum extensor moments in the upright position (ankle and knee) compared to the control and ET groups (*p* < 0.05).

#### 3.2.3. Music Therapy and Multiple Sclerosis

The effectiveness of MT in the clinical management of MS patients has been well investigated over the past few decades [37]. Maggio and colleagues (2021) evaluated the feasibility and effectiveness of a combined music therapy (MT) and exercise program for gait rehabilitation [38]. In this study, 20 patients were randomly assigned to either an MT group (*n* = 10) or a control group (*n* = 10). The control group received conventional therapy, while the MT group participated in an intervention program utilizing the Gait Trainer 3 (GT3) treadmill. The program was conducted three times per week for eight weeks, with each session lasting 30 min. The GT3 treadmill enhances traditional therapy by incorporating rhythmic auditory stimulation (RAS) and visual feedback. The device includes a library of metronome sounds and music compositions specifically designed by music therapists to optimize rhythm-based therapy. The RAS library integrates spatial, temporal, and force cues, promoting correct movement repetition and facilitating neuroplasticity through the retraining of existing neural pathways and the formation of new ones. This combined approach aids neurological patients in recovering motor function. All participants underwent evaluations at the beginning and end of the program. Motor assessments included the BBS, the TUG Test, and the 10-Minute Walk Test. The neurological assessment included the Beck Depression Inventory-II (BDI-II) and the MSQOL-54 to assess patients’ perception of their QoL, along with Goal Attainment Scaling (GAS). Additionally, participants completed the System Usability Scale (SUS) to assess the practicality of the intervention. By the end of the training program, the control group exhibited improvements in static and dynamic balance, as well as mood. However, the MT group showed significantly greater enhancements in static and dynamic balance, walking speed, mobility, mood, and QoL perception.

Impelizzeri and colleagues (2020) investigated the effects of MT on mood, motivation, emotional state, and cognitive functions in individuals with MS [39]. In this randomized controlled trial, 30 participants were assigned to either a control group (*n* = 15), which underwent conventional cognitive rehabilitation, or an MT group (*n* = 15), which received the same rehabilitation integrated with MT techniques. Both groups participated in six sessions per week for 8 weeks, with each session lasting approximately 60 min. Participants were assessed before and immediately after the intervention. Primary outcomes included cognitive function using a short and repeatable battery of neuropsychological (BRB-N) tests and QoL evaluation through the MSQoL-54 questionnaire. Additional measures included the Back Depression Inventory to measure mood and the Emotional Awareness Questionnaire (EAQ) to evaluate emotional processing and awareness. The results indicated that while both groups benefited from conventional rehabilitation, the MT group demonstrated significantly greater improvements in cognitive function, particularly in long-term storage, long-term retrieval, and delayed recall of the 10/36 spatial recall test. Furthermore, the MT group exhibited more pronounced enhancements in emotional status, motivation, mood, and QoL compared to the control group.

#### 3.2.4. Music Therapy and Other Neurological Disorders

Lee and colleagues (2024) explored the effectiveness of a combined MT and exercise program in children with ADHD [40]. The study involved 13 children who participated in a structured music and movement intervention for one hour per week over 8 weeks. During each session, participants learned to sing rhymes while performing specific body movements, guided by therapists who played live piano music. To reinforce the intervention, the parents facilitated daily at-home practice for 50 min. Participants were assessed before and after the program using multiple measures: the Paediatric Quality of Life Inventory (PedsQL) to evaluate QoL, the Kiddie Continuous Performance Test (K-CPT 2), and the Swanson, Nolan, and Pelham (SNAP-IV) Scale to assess ADHD symptoms, and elec electroencephalogram (EEG) recordings to examine neurophysiological changes. The results indicated significant improvement in the QoL of participants after the 8-week intervention. Additionally, reaction times in the K-CPT 2 were significantly reduced, indicating enhanced attentional control. EEG analysis revealed increased alpha power and Higuchi’s fractal dimension, along with a decrease in delta power in specific EEG channels, suggesting neurophysiological changes associated with improved cognitive function.

Lu and colleagues (2022) investigated the effectiveness of MT in managing sleep disorders in individuals with schizophrenia [41]. This randomized controlled trial included 66 participants, who were assigned to either an MT intervention group (*n* = 35) or a control group (*n* = 31). The TM group received a daily, one-hour music-based intervention for four weeks. The control group received standard care without music intervention. The Pittsburgh Sleep Quality Index (PSQI) was used to assess sleep disturbances, and the generalized estimating equation (GEE) analysis was employed to evaluate differences in PSQI score changes between the two groups from baseline to four weeks post-intervention. Results showed that the MT group experienced significantly greater improvements in sleep quality compared to the control group (group × time interaction; *p* < 0.001).

Cibrian and colleagues (2020) investigated the effectiveness of MT in improving coordination in children with autism spectrum disorder (ASD) [42]. In this randomized controlled trial, 22 children were assigned to either an experimental group (n = 11) or a control group (*n* = 11). Both groups participated in a weekly MT session for 8 weeks, engaging in exercises designed to improve bimanual coordination, strength regulation, and timing synchronization. The control group used traditional 10″ tambourines with wooden frames, while the MT group utilized BendableSound, an elastic touch display that allowed children to produce sounds by touching, tapping, or pinching a spandex fabric. Participants were assessed before and after the intervention using the Developmental Coordination Disorder Questionnaire (DCDQ) for coordination and the Playing in Touch (PiT) questionnaire to measure engagement in musical activities. Additionally, five tests evaluated timing synchronization and strength control. Results showed that all participants improved in coordination, as reflected in higher DCDQ scores, along with better movement control in post-intervention strength and timing assessments. Notably, children using BendableSound achieved significantly higher DCDQ scores than those using tambourines.

Moreu-Valls and colleagues (2025) evaluated the safety and efficacy of two cognitive rehabilitation strategies in individuals with early-to-middle-stage Huntington’s disease (HD) [43]. In this randomized controlled trial, 44 participants were assigned to one of three groups: an MT group (*n* = 16), a computerized cognitive training (CCT) group (n = 13), or a standard of care (SoC) group (*n* = 15). The MT and CCT groups participated in weekly sessions for 24 weeks, while the SoC group did not receive any active intervention.

The MT approach involved 45-min sessions conducted by a certified intervention. During each session, participants performed personalized MT cognitive exercises, including Auditory Perception Training, Musical Sensory Orientation Training, Musical Attention Control Training, Musical Executive Function Training, Musical Mnemonics Training, and Mood and Memory Association Training. Before the intervention, the CCT group participated in a 45-min computer-based session per week using the NeuronUp^®^ neurorehabilitation software (NeuronUP SL; https://neuronup.com). Each session included seven activities targeting five cognitive domains: executive functions, attention and processing speed, visuospatial skills, language, memory, and social cognition. The tasks were designed to increase difficulty progressively to maintain participant engagement and motivation throughout the intervention. Participants were evaluated at baseline and the end of the intervention program, with assessments including measures of global cognition, functional and motor skills, neuropsychiatric evaluations, and structural and functional neuroimaging. The results showed significant improvements in overall cognition and disease severity in both the MT and CCT groups. These interventions not only enhanced cognitive function but also led to structural and functional changes in critical brain areas associated with HD. More information about MT in different neurological disorders can be found in Table 2.

## 4. Discussion

This narrative review explores the effectiveness of TC and MT in the treatment of neurological disorders, highlighting their potential benefits for individuals. The findings suggest that TC significantly improves physical function, balance, and QoL and reduces fall risk, while MT enhances cognitive function, motor performance, and psychological well-being.

Unlike conventional training, which typically takes place in dedicated facilities with direct interaction between trainers and participants, TC leverages digital tools and technologies to remotely manage and deliver training programs [8]. Common TC strategies include the use of wearable devices, mobile applications, and VR systems, often enhanced by gaming consoles. Wearable devices, such as smartwatches, play a crucial role in continuous and non-invasive monitoring of key health data, including heart rate, daily step count, walking speed, and weekly physical activity levels [44,45].

Applications and digital platforms further enhance TC by enabling the creation of personalized exercise plans and facilitating the remote delivery of training programs aimed at improving physical activity and cardiorespiratory fitness [46,47]. One particularly effective TC strategy is VR-based training, which immerses participants in interactive, simulated environments that mimic real-life activities. This approach not only increases physical activity levels but also enhances executive function, short-term memory, and long-term memory [48,49]. Moreover, VR-based TC improves participant engagement by dynamically adjusting difficulty levels based on individual skill levels, helping overcome barriers such as time constraints, geographical distances, and transportation challenges [8].

The effectiveness of TC has been demonstrated across various populations, including older adults [50,51] and individuals with respiratory [52,53], metabolic [54], and cardiovascular diseases [55]. However, further research is needed to precisely determine its long-term effectiveness and clarify its role as both a preventive and therapeutic approach [56,57].

The mechanisms through which TC influences neurological disorders are multifactorial. Firstly, TC provides personalized and flexible interactions, which enhance motivation and adherence to the training programs. Continuous engagement with a coach can strengthen participants’ commitment to rehabilitation, fostering a sense of responsibility for their progress. Additionally, TC develops practical and cognitive skills, improving awareness and decision-making related to well-being [58].

Beyond behavioural benefits, TC actively stimulates neuroplasticity, potentially aiding in the recovery of motor and cognitive functions [59]. Research shows that the human brain is highly adaptable and capable of reorganizing itself in response to new demands and environmental influences [60]. Neuroplasticity plays a key role in acquiring new skills and recovering from neurological injuries [61]. Similarly, MT is believed to leverage neuroplasticity. Whether through active participation or passive listening, MT engages multiple neural pathways and sensory modalities in the brain. Structural changes occur at both cortical and subcortical levels, impacting regions responsible for sensorimotor functions [62], auditory perception [63], and white-matter tracts [64]. Additionally, MT has been associated with increased grey matter density in the left anterior hippocampus, a region linked to memory and learning [65].

Another key mechanism of MT is its influence on psychological and social processes. Music listening is strongly associated with stress reduction, as it lowers physiological arousal in cortisol levels, heart rate, and blood pressure [66,67]. MT also reduces negative emotions such as worry, anxiety, and nervousness [68,69]. Recent studies suggest that music modulates brain structures like the amygdala and the mesolimbic system, both of which are crucial for emotional regulation and motivation.

Moreover, group-based MT fosters social connections. The synchronization of movements and rhythms during MT sessions strengthens interpersonal bonds, generating positive feelings among participants [70]. These effects may be driven by the release of neurotransmitters such as endorphins and oxytocin, which play a central role in stress regulation, emotional bonding, and social cohesion [71,72].

The effectiveness of MT can also be measured in terms of its impact on cognitive functions, particularly memory. A recent study by Moreira and colleagues (2023) explored the effects of MT on memory decline in the elderly [73]. In this randomized, double-blinded, controlled study, 42 elderly participants received MT sessions led by a certified music therapist. The results indicated that structured MT interventions can effectively reduce cognitive decline, benefiting both elderly individuals with and without cognitive impairment. This highlights the potential of MT to enhance neuroplasticity and cognitive functioning, further indicating its value as a therapeutic tool. These findings suggest that MT may not only improve emotional and social well-being but also play a critical role in cognitive rehabilitation, particularly in individuals at risk for memory decline.

However, the potential limitations and negative effects of these innovative approaches must also be considered. Regarding TC, the main limitations may include low technological ability among some patients and the possible lack of a stable internet connection. These factors could negatively impact patient engagement and reduce adherence to the intervention program. For music therapy, physical limitations may reduce the ability of neurological patients to actively participate in musical activities, and the shortage of qualified therapists may limit the effectiveness of the treatment.

An additional factor to consider when implementing these interventions is the influence of circadian rhythms on both physical and cognitive performance [74]. Circadian rhythms impact not only sleep but also mood, emotional responses, and physical abilities [75,76]. For individuals with neurological disorders, who often experience fatigue and cognitive fluctuations, it is important to schedule therapy sessions during optimal times of day when they are most energetic and alert [77]. Aligning TC and MT interventions with circadian rhythms may also improve sleep quality, which is frequently compromised in these populations [78]. In this context, our review findings suggest that while MT can enhance mood, attention, and QoL in individuals with PD, it does not lead to improvements in dual-task performance or balance ability. The progression of the disease may limit neuronal plasticity, preventing long-term effects on motor and cognitive abilities in this population. Cognitive and motor functions in this condition may require more specific and multi-component interventions to achieve substantial improvements [79]. In addition, the role of patient demographics and the presence of comorbidities may affect the effectiveness of the intervention [80]. Similarly, in patients with MS, the PBCST program has been shown to be more effective when delivered through a traditional method rather than a TC approach. The effectiveness of PBCST may be enhanced by direct interaction with a therapist, highlighting the importance of supervised training in some conditions. Therefore, it is essential to adapt therapeutic approaches to meet the specific needs of the population. Administering these interventions at times when individuals feel most motivated and energized could significantly enhance their effectiveness [76].

### 4.1. Proposed Integrated Intervention Program: Music Therapy and Telecoaching for Neurological Disorders

This proposal presents an integrated program that combines MT and TC to enhance the physical, cognitive, and social well-being of individuals with neurological disorders.

The selection of participants for the integrated TC and MT program will be conducted through a rigorous screening process that employs the following inclusion criteria: (a) a confirmed diagnosis of a neurological disorder; (b) age between 18 and 75 years; (c) readiness to engage in a 12-week training program; and (d) signed informed consent.

The evaluation of the training program will involve both instrumental assessments and questionnaires specifically designed to assess various aspects of neurological disorders. The assessments will include the State-Trait Anxiety Inventory (STAI) to evaluate emotional function, the BBS and TUG Test to assess balance and fall risk, the Pittsburgh Sleep Quality Index to measure sleep disturbances, the 36-Item Short Form Survey Instrument (SF-36) to evaluate QoL, and the International Physical Activity Questionnaire (IPAQ) to assess levels of physical activity. These assessments will enable the measurement of changes in physical functioning, cognitive abilities, and emotional well-being throughout the duration of the intervention.

The intervention would consist of weekly TC sessions led by sports and exercise science professionals, guiding participants through personalized physical activities. Participants would receive tailored training programs supported by wearable devices that monitor their goals and make necessary adjustments to their training.

Additionally, the training program would utilize video calls and dedicated platforms to facilitate ongoing communication between participants and professionals. This approach fosters an engaging and supportive environment, encouraging participants to remain motivated and connected. These strategies will be effective in addressing potential variability in participants’ engagement, enabling the collection of objective data and facilitating personalized adjustments to training programs based on individual needs.

MT sessions could be conducted online by certified music therapists and would include a variety of activities such as active listening, passive listening, musical improvisation, and group activities. These sessions aim to promote social interaction and the release of positive emotions.

The intervention program could last for 12 weeks, with three sessions per week and continuous monitoring. It should commence with an assessment of the participant’s physical and cognitive condition using standardized tests and questionnaires.

A combined program of TC and MT promotes continuous monitoring, supports the development of a sustainable intervention, enhances motivation and adherence, fosters stronger social relationships, and leads to improvements in cognitive functions and memory.

In summary, these integrated TC and MT programs represent a valuable opportunity to enhance well-being among individuals with neurological disorders. This combined approach aims to address cognitive and physical decline while providing emotional support, ultimately contributing to an improved QoL.

### 4.2. Strength and Limitations

One of the primary strengths of this study is its comprehensive examination of TC and MT in the treatment of neurological disorders. The review provides valuable insights into the benefits of both approaches, highlighting potential physical, cognitive, and emotional improvements for patients.

Another significant strength is the exploration of the mechanisms of action for both approaches. Understanding these mechanisms, particularly the role of neuroplasticity, establishes a solid theoretical foundation for their application in treatment.

Additionally, in today’s digital age, the incorporation of technology and wearable devices is an important aspect to consider. These tools help overcome barriers by enhancing motivation, monitoring progress, and ensuring high adherence to intervention programs.

However, this study has some limitations. Firstly, as it is a narrative review rather than a systematic review, there was no rigorous methodology for selecting studies, which could introduce bias in the results. Additionally, the variability in intervention programs, along with differences in administration methods and duration, complicates the comparison of results. Finally, while the review analyses the potential impacts of the TC and MT on cognitive, psychological, and emotional well-being, the lack of long-term follow-up limits the ability to comprehensively evaluate the effects of these approaches over time.

## 5. Conclusions

In conclusion, the findings of this review suggest that TC and MT are effective alternative approaches for treating neurological disorders. Both TC and MT, as well as their combination, enhance physical well-being while also improving the overall QoL by enhancing cognitive abilities and psychological well-being in individuals with neurological disorders. Neuroplasticity emerges as a key mechanism through which these approaches positively influence brain functions. However, the limited number of studies assessing long-term effects highlights the need for further research to explore these interventions in greater depth and to develop practical guidelines for their implementation in clinical practice.

## Figures and Tables

**Table 1:** Telecoaching Approach in Different Neurological Disorders.

	Neurological Disorder	TC Strategies	Main Results
Hoang, 2015 [24]	MS	Stepmania open source software; Supervised training sessions; Asynchronous TC approach; Interactive exergaming systems; Weekly progress assessment calls.	TC training program is a feasible, safe, and effective approach for improving stepping, standing balance, coordination, and functional performance in individuals with MS.
Yang, 2016 [21]	PD	Virtual reality balance training; Synchronous TC approach.	TC and conventional balance training are equally effective in enhancing balance, gait, and QoL in individuals with PD.
Gandolfi, 2017 [20]	PD	Virtual reality balance training; Supervised training sessions; Synchronous TC approach; Interactive exergaming systems. Weekly progress assessment calls; Logbook.	TC is a viable and cost-effective alternative to conventional training for reducing postural instability in individuals with PD.
Burns, 2017 [28]	CMT	Supervised training sessions; Asynchronous TC approach; Digital tools.	TC resistance training attenuates the long-term progression of dorsiflexion weakness without adverse effects in children with CMT disease.
Atterbury, 2017 [22]	PD	Asynchronous TC approach; Digital tools.	TC is effective in improving some aspects of gait, such as stride velocity and cadence, in individuals with mild to moderate PD.
Pagliari, 2021 [23]	MS	Virtual reality training; Asynchronous TC approach; Offline Remote Monitoring.	TC is effective in improving QoL and alleviating motor symptoms in individuals with MS.
Mehta, 2021 [29]	MG	Supervised training sessions; Asynchronous TC approach; Weekly progress assessment calls; Digital tools; Logbook.	TC approach, when added to standard care, improves QoL and reduces headache frequency in individuals with MG.
Güngör, 2022 [25]	MS	Supervised training sessions; Asynchronous TC approach; Weekly progress assessment calls.	TC approach can be recommended for individuals with limitations in attending supervised sessions to improve postural control, agility, and strength in MS.
Menengi, 2022 [30]	AD	Synchronous TC approach; Digital tools.	TC dual-task training programs can lead to significant improvements in cognition and mobility, enhance functional independence, and reduce symptoms of anxiety and depression in individuals with AD.
Jabri, 2022 [27]	AT	Digital tools; Synchronous TC approach.	TC balance and coordination training program improves physical performance in individuals with AT.
Cabanas Valdés, 2024 [26]	AT	Supervised training sessions; Asynchronous TC approach; Weekly progress assessment calls. Digital tools; Logbook.	TC improves balance confidence, gait speed, and QoL and reduces the fall rate in individuals with AT.

Legend: (TC) Telecoaching; (PD) Parkinson’s disease; (MS) Multiple Sclerosis; (CMT) Charcot–Marie–Tooth; (MG) Migraine; (AD) Alzheimer’s disease; (AT) ataxia.

**Table 2:** Music Therapy Approach in Different Neurological Disorders.

	Neurological Disorder	MT Strategies	Main Results
Gómez Gallego, 2017 [31]	AD	Comfortable, well-lit, and soundproof rooms; Varied musical activities; Music played through speakers.	MT is effective in improving various cognitive, psychological, and behavioural alterations in individuals with AD.
Lyu, 2018 [33]	AD	Familiar and favourite songs.	MT is effective in enhancing cognitive function and mental well-being in individuals with AD.
Pohl, 2020 [34]	PD	Ronnie Gardiner Method; Varied musical activities; Music played through speakers.	MT is effective in improving the QoL in individuals with PD; however, it does not lead to improvements in dual-task performance, cognition, or balance.
Cibrian 2020, [42]	ASD	BendableSound; 10” tambourines with a wood frame; Familiar and favourite songs.	MT is effective in improving the coordination skills of children with ASD.
Impelizzeri, 2020 [39]	MS	Associative Mood and Memory Training (AMMT); Music in Psychosocial Training and Counseling (MPC).	MT is an effective complementary approach to conventional rehabilitation in individuals with MS.
Gómez Gallego, 2021 [32]	AD	Comfortable and spacious rooms; Familiar and favourite songs.	MT is effective in improving AD symptoms and could be prescribed as a complementary treatment alongside standard care.
Fodor, 2021 [35]	PD	Familiar and favourite songs.	MT combined with a multimodal program focused on physical therapy may be beneficial for individuals with PD.
Maggio, 2021 [38]	MS	Rhythmic auditory stimulation (RAS).	The integration of the GT3 with MT is a feasible and effective approach for gait rehabilitation in individuals with MS.
Lu, 2022 [41]	SH	Music with a slow beat of 60–80 per minute; Familiar and favourite songs.	MT is effective in reducing sleep disturbances among individuals with SH.
Li, 2022 [36]	PD	Headphone audio playback; Familiar and favourite songs.	MT associated with exercise is effective in improving gait disorders in individuals with PD.
Lee, 2024 [40]	ADHD	Music played on a piano; Familiar nursery rhymes.	MT combined with exercise is an effective and alternative approach for treating ADHD in children.
Moreu-Valls, 2025 [43]	HD	Varied musical activities; Familiar and favourite songs.	MT is effective in improving cognitive function and the severity of the disease in individuals with HD.

Legend: (MT) Music Therapy; (AD) Alzheimer’s disease; (PD) Parkinson’s disease; (ASD) autism spectrum disorder (MS); Multiple Sclerosis; (SH) schizophrenia; (ADHD) Attention-deficit/hyperactivity disorder; (HD) Huntington’s disease; (QoL) Quality of Life.
